# The effect of navigation method and visual display on distance perception in a large-scale virtual building

**DOI:** 10.1007/s10339-020-01011-4

**Published:** 2021-02-09

**Authors:** Hengshan Li, Panagiotis Mavros, Jakub Krukar, Christoph Hölscher

**Affiliations:** 1 Future Cities Laboratory, Singapore-ETH Centre, 1 CREATE Way, CREATE Tower, 138602 Singapore, Singapore; 2 Institute for Geoinformatics, University of Muenster, Münster, Germany; 3 Department of Humanities, Social and Political Sciences, ETH Zürich, Zurich, Switzerland

**Keywords:** Distance judgements, Perceived distance, Traversed distance, Environmental distance, Optic flow, Movement interfaces

## Abstract

Immersive virtual reality (VR) technology has become a popular method for fundamental and applied spatial cognition research. One challenge researchers face is emulating walking in a large-scale virtual space although the user is in fact in a small physical space. To address this, a variety of movement interfaces in VR have been proposed, from traditional joysticks to teleportation and omnidirectional treadmills. These movement methods tap into different mental processes of spatial learning during navigation, but their impacts on distance perception remain unclear. In this paper, we investigated the role of visual display, proprioception, and optic flow on distance perception in a large-scale building by manipulating four different movement methods. Eighty participants either walked in a real building, or moved through its virtual replica using one of three movement methods: VR-treadmill, VR-touchpad, and VR-teleportation. Results revealed that, first, visual display played a major role in both perceived and traversed distance estimates but did not impact environmental distance estimates. Second, proprioception and optic flow did not impact the overall accuracy of distance perception, but having only an intermittent optic flow (in the VR-teleportation movement method) impaired the precision of traversed distance estimates. In conclusion, movement method plays a significant role in distance perception but does not impact the configurational knowledge learned in a large-scale real and virtual building, and the VR-touchpad movement method provides an effective interface for navigation in VR.

## Introduction

Understanding human distance perception while learning a new large-scale environment is important in explaining and modeling spatial learning and wayfinding behaviors. Most navigators begin to acquire metric and configurational knowledge (i.e., distance and direction) on first exposure to a new environment, which improves over time (Ishikawa and Montello 2006; Montello 1998). This spatial knowledge acquisition process often involves the encoding of distance between different objects and locations, which requires the integration of perceived distance, traversed distance, and environmental distance (Loomis et al. 1996; Montello 1997; Sadalla and Staplin 1980). However, distance perception of large-scale environments is subject to different external and internal sources of bias. It can be affected by environmental features, such as stimulus orientation, available depth cues, intersections of a route and others (Da Silva 2006; Sadalla and Staplin 1980; Waller 1999; Wiest and Bell 1985) as well as internal sensory and cognitive processes, such as vestibular and proprioceptive feedback or optical velocities (Bremmer and Lappe 1999; Loomis et al. 1999b; Witmer and Kline 1998).

During the last decades, virtual reality (VR) is becoming increasingly common for the study of spatial behavior and cognition, as well as for other types of research, such as pre-occupancy building evaluations (Shin et al. 2017) or evacuation and safety (Kinateder et al. 2014, 2018). In order to reduce the frequently occurring issue of VR-induced motion-sickness, different approaches are used to emulate movement in VR, which include, among others: continuous translation, intermittent translation, teleportation, or the use of omnidirectional treadmills to provide proprioceptive feedback. However, it is yet unclear how these approaches influence perceptions of space and whether they are source of bias in the perception of distance.

In this context, we designed the present study with three primary goals. First, to investigate whether distance perception (verbally reported) is influenced by experiencing space in VR (i.e., effect of visual display). We tested this by comparing distance perception of a large-scale building in the real-world and in its replica in VR. Second, we were interested on the effects of proprioception and continuity of optic flow on traversed distance and on environmental distance estimates. To test this, we implemented three methods for movement in VR (see Table 1) including (a) walking on an VR omnidirectional treadmill (continuous visual flow and proprioceptive feedback), (b) touchpad controller (continuous visual flow and no proprioceptive feedback), and (c) VR teleportation (intermittent visual flow and no proprioceptive feedback). Finally, we investigated whether the accuracy of participants’ distance judgements is consistent across environmental, perceived, and traversed distances.

**Table 1:** Sensory inputs of different movement methods

Sensory input	Experimental conditions			
	Walking	Treadmill walking	Touchpad	Teleportation
Visual display	Real world	VR	VR	VR
Proprioception	Present	Present	Absent	Absent
Optic flow	Continuous	Continuous	Continuous	Intermittent

VR-treadmill provides both proprioception and continuous optic flow, VR-touchpad allows for continuous optic flow, and VR-teleportation only allows for intermittent optic flow

This article is structured as follows: first we discussed relevant previous research and how it relates or motivates our research goals. Second, we introduce the methods, including the experiment design and analysis plan. When present the results of the statistical analyses and proceed with their discussion in the context of previous and future research.

## Background

### Types of distance

#### Perceived distance

Perceived distance refers to the apparent distance between the observer and a stimulus in vista space that is immediately perceivable (Baird 1970; Foley 1980; Montello 1997). Psychophysical research has previously focused on modeling perceived distance in the real world based on Stevens’ power law *Y* = *kX*^*n*^ (Stevens and Galanter 1957). The modulus *k* represents a constant defining the scale unit, and the exponent *n* reflects the acceleration of the function between actual distances (*X*) and estimated distances (*Y*). When *n* equals 1, perceived distance is a linear function of actual distance; when *n* is larger (or smaller) than 1, there is a positive (or negative) acceleration of the power function. For a natural setting, a wide range of the exponent *n* (from about 0.6 to 1.5) has been observed for verbal report of perceived distances, as distance estimates are sensitive to estimation tasks and environmental settings (for a review, see Da Silva 2006). For indoor settings, a large body of previous literature has yielded mixed results (Lappin et al. 2006; Philbeck et al. 2018; Philbeck and Loomis 1997). Some studies have shown that the exponent n is often larger than 1 (ranging from about 1.2 to 1.5), indicating that people tend to overestimate perceived distances in a natural indoor setting (Künnapas 1960; Luria et al. 1967; Teghtsoonian and Teghtsoonian 1969). Other studies, however, have shown that perceived distances can be estimated accurately mostly using laboratory settings (i.e., small-scale environments). For example, Loomis et al. (1996) reported that no large systematic error was observed when blindfolded observers walked to a previously seen target in a well-lit environment.

#### Traversed distance

Traversed distance refers to the length of a route covered during movement (Sadalla and Magel 1980; Sadalla and Staplin 1980). During movement, navigators rely on external signals (i.e., visual and acoustic flow) and internal signals (i.e., proprioception and vestibular feedback) to estimate speed of movement and update self-position and orientation with respect to the start location (see Loomis et al. 1999b for the review of path integration). While perceived distance primarily relies on visual cues, traversed distance requires the integration and memory of spatial information over time, thereby it can be affected by visual, cognitive, and proprioceptive cues (Heft 1996; Proffitt et al. 2003; Sadalla and Magel 1980; Witmer and Kline 1998). Previous literature has found that walking speed (Elliott 1987), number of turns of a route (Sadalla and Magel 1980), and intersections along a route (Sadalla and Staplin 1980) affect the estimation of traversed distance. For example, Sadalla and Staplin (1980) found that participants consistently perceived a route with more intersections longer than an equivalent-length route with fewer intersections, even though travelled time for both routes were approximately the same. ﻿

#### Environmental distance

Environmental distance is the straight-line distance (i.e., Euclidean distance) between two separate places in a large-scale environment (Montello 1993) that “cannot be perceived from a single vantage point but requires movement through the environment for its apprehension” (Montello 1997). For a large-scale built environment, interior objects such as walls, ceilings, and other obstacles often block visual access between places. In order to perceive the entire layout, navigators have to move through the space and integrate separately learned places into a globally coherent mental representation, which is often referred to as a cognitive map (Tolman 1948). Accordingly, environmental distance was previously called cognitive distance (Montello 1991), although both perceived and traversed distance require perceptual and cognitive processes (Montello 1997). For this reason, in this study we used the term of environmental distance.

Here, it is useful to distinguish between egocentric and allocentric spatial reference frames (see Klatzky 1998; Mou et al. 2004; Mou and McNamara 2002). In the egocentric reference frame, the location and orientation of objects are organized with respect to the observer, whereas in the allocentric reference frame, the location and orientation of objects are specified with respect to the environment. Thus, perceived and traversed distance are egocentric, whereas environmental distance is allocentric. Previous literature on representational flexibility has found that navigators acquire allocentric and egocentric spatial knowledge in parallel (Brunyé et al. 2008; Iglói et al. 2009) and that both learning perspective and learning goal (as well as individual differences) influence cognitive map development (Meilinger et al. 2016; Pazzaglia and Taylor 2007). In an outdoor setting, Ishikawa and Montello (2006) found that, after participants built more accurate metric knowledge of the space by integrating separately learned routes, traversed distance estimates were not improved. However, to our best knowledge no empirical evidence has been found about whether the accuracy of learning the entire layout (i.e., global allocentric knowledge) of a large-scale indoor environment is associated with accuracies of traversed and perceived distance judgements on different parts of the building (i.e., local egocentric knowledge).

### Factors influencing distance perception

Research has identified multiple factors that influence distance perception, including external factors such as visibility (and lighting), space shape and typology, indoor vs outdoor spaces, as well as internal factors such as proprioception and vestibular feedback. For example, although Loomis et al. (1996) reported no large systematic error when blindfolded observers walked to a previously seen target in a well-lit small-scale environment, a subsequent study under dim-lighting condition resulted in overestimation of proximal targets and underestimation of distal targets (Philbeck and Loomis 1997). Other recent studies have found that the environmental context contributed to the mixed results of perceived distance estimation (Iosa et al. 2012; Lappin et al. 2006; Philbeck et al. 2018; Witt et al. 2007). For instance, Lappin et al. (2006) found that environmental context (e.g., indoor hallway, indoor lobby, and outdoor lawn) affected the accuracy of perceived distance; participants overestimated distances in an outdoor space compared to an indoor lobby. Witt et al. (2007) found that perceived distance judgments were overestimated when targets were seen at the shorter end of a long indoor hallway than the longer end of the same hallway. More recently, Philbeck et al. (2018) found that the response mode of distance estimates impacted the environmental context effects such that verbal and size gesture judgments showed no context effects, whereas blindfolded-walking responses were shorter indoors than outdoors.

The contribution of internal factors on perception of distance has also been of interest to researchers. Although perceived distance primarily relies on visual cues (Heft 1996; Sadalla and Staplin 1980; Witmer and Kline 1998), research on embodied cognition suggests that visual processing is influenced by a person’s top-down processes including purpose, physiological state and emotions (Proffitt 2006a, b; Proffitt et al. 2003). Santillán and Barraza (2019) have found that motor proprioceptive information and optic flow also played a role in perceived distance estimate. Despite the extensiveness of previous literature, the role of internal signals played in spatial learning is still debated (for a review, see Chrastil and Warren 2012). Some studies report that internal signals play a minor role in spatial learning and have found that both vestibular and proprioceptive feedback did not affect traversed distance estimates (Bremmer and Lappe 1999; Richardson et al. 1999; Witmer and Kline 1998). For example, Witmer and Kline (1998) compared traversed distance estimate in a virtual hallway learned via different movement methods including treadmill, joystick, and teleportation. They found that proprioception did not affect traversed distance estimates, as the treadmill group performed no better than the joystick or teleportation groups. Other studies, however, reported that idiothetic information (including motor and proprioceptive and vestibular information) contributes to path integration and spatial learning in large-scale environments (Campos et al. 2012; Ruddle et al. 2011; see Chrastil and Warren 2012 for a review). For example, Campos et al. (2012) found that both proprioceptive and vestibular cues contribute to travelled distance estimates during walking in a small-scale (12 m × 15 m) free-walking space. As a result, more research is needed to disentangle the role of body-based sensory information in distance perception. Furthermore, given that the majority of these studies have been conducted in small-scale spaces, such laboratories or rooms, it is important to study the perceptual and cognitive processes of distance perception in larger and more complex environments.

### Distance perception research in VR

Recent advances in computing power, computer graphics technology, and a new generation of higher resolution head-mounted displays have led VR to become a common tool used to investigate perception and cognition (Kinateder et al. 2018; Knapp and Loomis 2003; Loomis et al. 1999a; Moussaïd et al. 2018; Renner et al. 2013; Creem-Regehr et al. 2015b; Kelly et al. 2017; Siegel et al. 2017). Earlier research has found that people can develop accurate survey knowledge of large-scale virtual environments (in terms of distance and direction between targets), although the learning process in VR typically takes a longer time than in physical buildings (e.g., Richardson et al. 1999; Ruddle et al. 1997). On the other hand, a large body of studies using a variety of environmental conditions have found that people tend to underestimate perceived distances in VR compared to actual distances (Creem-Regehr et al. 2015a; Renner et al. 2013; Witmer and Kline 1998; Kelly et al. 2017).

However, many of the earlier studies discussed above used older-generation HMDs with lower visual resolution, which may impact the perception of visual flow which influenced both by pixel density and frame rate. The new generation of HMDs and game-engines affords better visual fidelity, which could potentially improve spatial perception in general. In addition, although new VR navigation modalities, such as teleportation or omnidirectional treadmill, directly influence visual flow and proprioceptive input, respectively, it is not yet clear how they impact perception of space and distance in VR.

Indeed, researchers have started to disentangle the impacts of visual and proprioceptive feedback on traversed distance estimates in VR (Campos et al. 2012; Moussaïd et al. 2018; Nescher et al. 2014; Waller et al. 2007). For instance, the teleportation method has been recently adopted in VR applications due to the ease of use and low motion sickness (Bhandari et al. 2018; Cherep et al. 2020; Christou and Aristidou 2017; Langbehn et al. 2018; Mayor et al. 2019; Moghadam et al. 2020). However, teleportation does not provide continuous optic flow during movement. Previous literature has shown that optic flow plays an important role in path integration, spatial updating, and spatial learning (for a review see Chrastil and Warren 2012; Kearns et al. 2002). Indeed, it has been found that the teleportation interface impaired spatial awareness (Moghadam et al. 2020), spatial updating (i.e., updating self-location during travel; Cherep et al. 2020), and spatial orientation (Bhandari et al. 2018). Similarly, with regard to proprioception, Ruddle et al. (2011) found that participants who walked using a treadmill (either omnidirectional or linear) had built more accurate survey knowledge, compared to participants who used a joystick for navigation, in a large-scale environment.

Another limitation many of the studies discussed above have in common is the use of small-scale environments as stimuli (e.g., empty rooms, green fields, etc.). The distinction between large- and small-scale is particularly important when investigating the effects of body-based sensory information (i.e., proprioception and vestibular feedback) on traversed distance estimate, as the body-based sensory information is used in path integration (i.e., the updating of position and heading on the basis of velocity and acceleration information), and path integration errors increase with spatial scale (Loomis et al. 1993, 1999b). A large-scale environmental space has to be perceived by moving through the space, whereas the entire spatial extent of a small-scale vista space can be seen from a single vantage point with head rotation (Montello 1993). A large-scale public indoor space such as a shopping mall not only has a larger extent but also contains more complex physical and social features such as lobby, atrium, and crowds, compared to a small-scale indoor setting. Previous literature has found mixed results on the effect of distance range on perceived distance estimate (Da Silva 2006; Nguyen et al. 2011), suggesting that people might perform differently between a small- and large-scale space. In addition, spatial abilities at different scales have been found to be partially dissociable (Hegarty et al. 2006). Given that environmental context has been found to play an important role in distance perception (Lappin et al. 2006; Philbeck et al. 2018; Witt et al. 2007) and that most research on human wayfinding investigates our ability to perform tasks in large-scale spaces, it is important to study the difference of distance perception and spatial cognition between a large-scale spaces.

### The present study

The study aims to examine the effects of visual display, proprioception, and the continuity of optic flow on perceived, traversed, and environmental distance estimates. Toward this end, we tested the following hypotheses based on the previous discussion:

H1: Visual display should affect the estimations of perceived (H1.1) and traversed distances (H1.2) but not affect environmental distances (H1.3) in a large-scale public indoor space
H2: Proprioception should not affect the estimations of perceived (H2.1) and traversed distances (H2.2) but affect environmental distances (H2.3) in a large-scale public indoor space
H3: The continuity of optic flow should not affect the estimations of perceived (H3.1) and traversed distances (H3.2) but affect environmental distances (H3.3) in a large-scale public indoor space
H4: Perceived distances in a large-scale public indoor space (exploratory analyses were conducted for traversed distances) should be underestimated in VR but overestimated in the real world, compared to actual distances
H5: There should be a positive acceleration of the power function (*n* >1) between actual distances and perceived distance estimates in the real world (exploratory analyses were conducted for the VR conditions)
H6: The accuracy of perceived and traversed distance estimates in a large-scale public indoor space should not be associated with the accuracy of environmental distance estimates

## Methods

### Participants

A total of 82 participants were recruited from universities in Singapore. The main inclusion criteria were that participants were unfamiliar with the testing environment (Westgate Shopping Mall, Jurong East, Singapore) and had normal or corrected-to-normal vision. Two people did not finish the experiment due to simulator sickness and were not included in the analyses. The final sample included 80 participants (40 females; mean age = 22.1 years; age range = 18 to 35). All participants completed an informed consent form before the study. Participants required approximately 50 min to complete the task and were compensated 20 Singaporean dollars for their participation. The study was approved by the Research Ethics Committee of ETH Zurich (2016-N-73).

### Materials

**Fig. 1:**
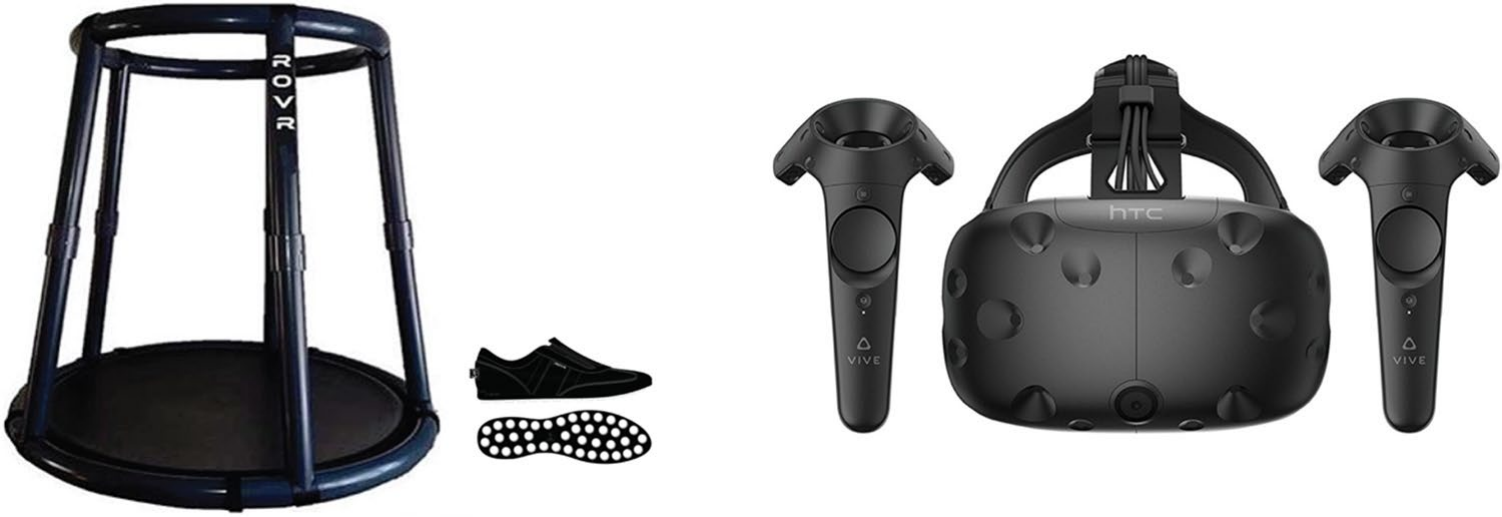
The ROVR omnidirectional treadmill and the special low friction shoes (left). An HTC Vive HMD and two controllers (right)

The VR setup consisted of a desktop computer, an HTC Vive HMD (2017 version), a handheld HTC Vive Controller, and an omnidirectional ROVR VR-treadmill (Wizdish Ltd; see Fig. 1). The desktop computer was equipped with an Intel Core i7-6700 K processor (3.40 GHz) and ran Windows 10 Enterprise with a GeForce GTX 1080 graphics card. The HMD had 360° head-tracking with a 110° field of view, 1080 × 1200 pixels resolution (per eye), and a refresh rate of 90 Hz.

The software for the VR setup included Unity (Unity Technologies) and Steam VR (Valve Corporation) for virtual environments rendering and interaction control. The virtual environments were created based on the architectural drawings of Westgate Shopping Mall in Singapore using 3DsMax (Autodesk, Inc.).

#### The testing environment

**Fig. 2:**
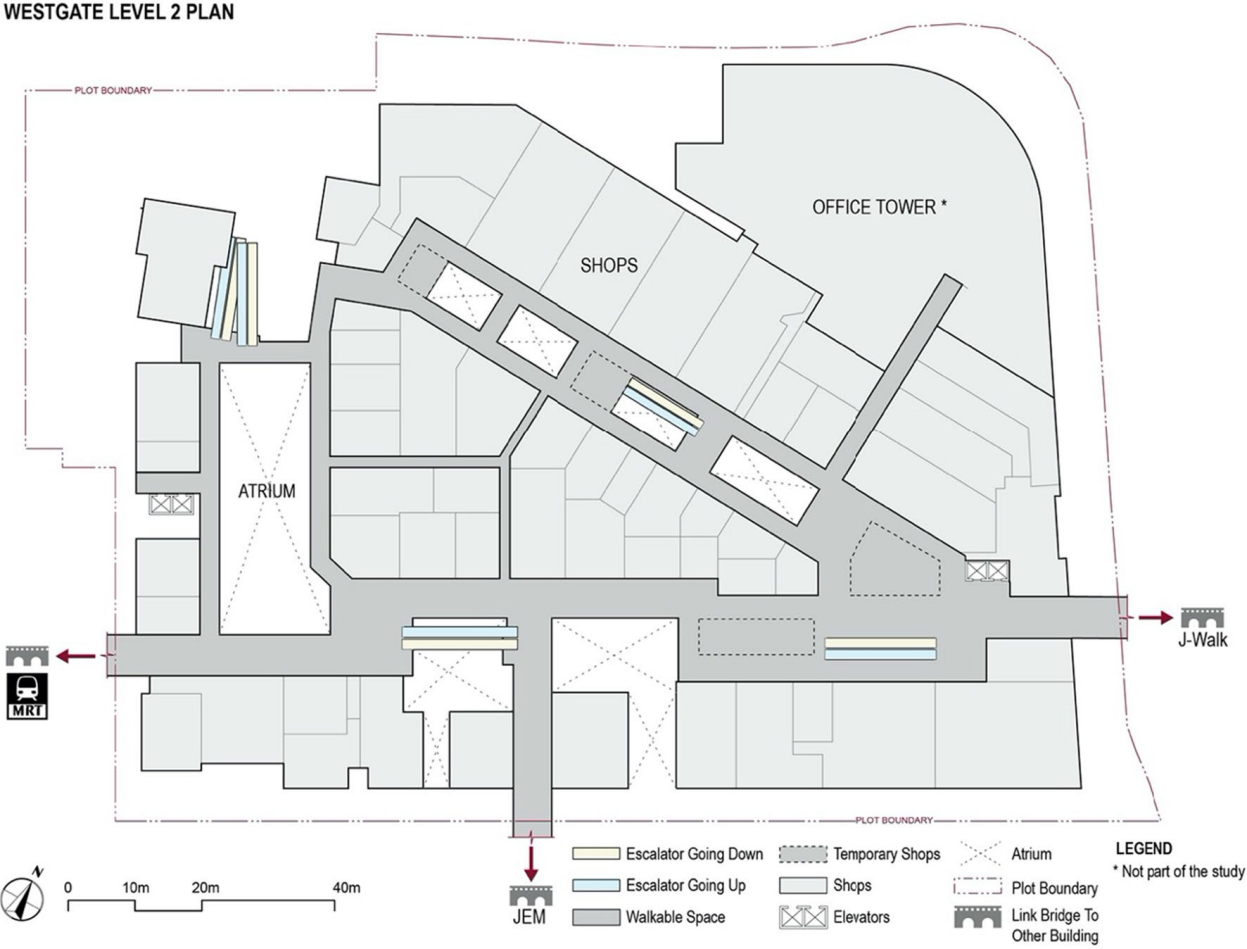
The layout of the Westgate Shopping Mall (Floor 2) in Singapore

**Fig. 3:**
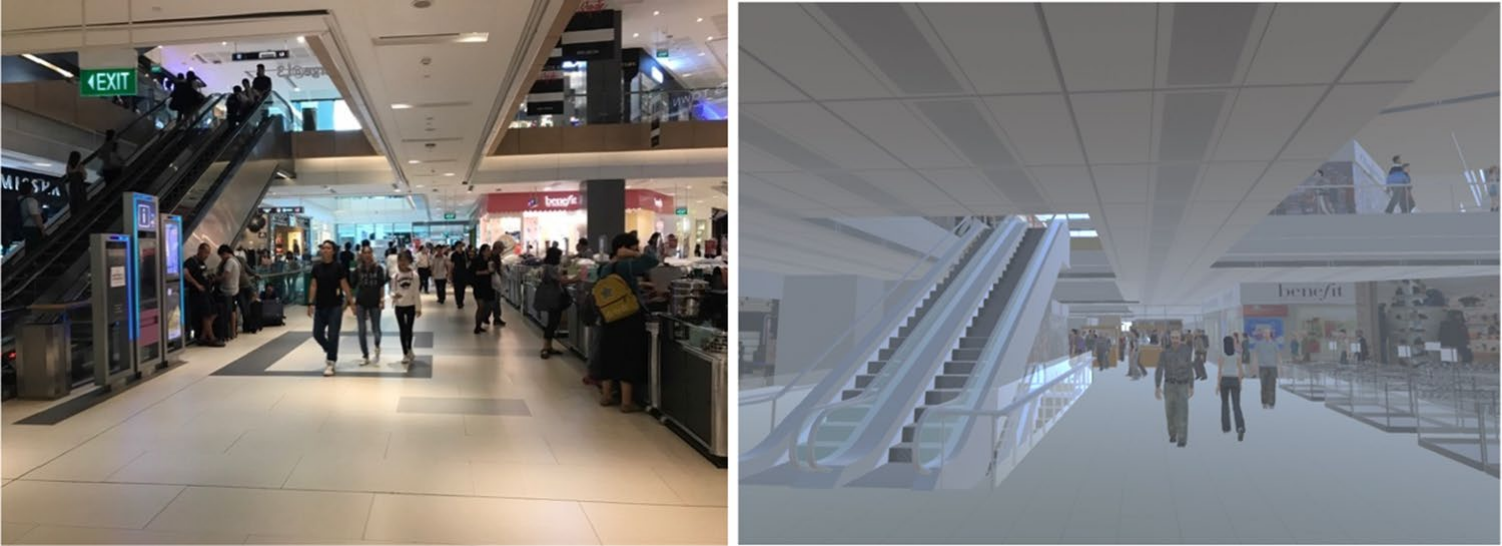
Both the real world (left) and the virtual replica (right) of the Westgate shopping mall were populated with crowds (for details of crowds simulation, see Li et al. 2019)

The experiment was conducted in either the real or the virtual replica of the Westgate Shopping mall. Westgate is a large building measuring 141 × 108 meters, and each floor has an approximate surface area of 13,430 sqm (including open spaces and atria). The real mall consisted of seven levels, but in the present study we used exclusively the second floor, which consists of approximately 2964 sqm of walkable spaces (see Fig. 2). Notably, there were pedestrians moving through the real-world building during the study (see Fig. 3). To emulate the potential effect of pedestrian crowds on distance perception, we designed and simulated virtual crowds in VR (see Fig. 3). In this manner, participants both in the real world and in VR experienced the building occupied with crowds.

#### Movement methods

In the real world, participants directly walked in the building. In VR, participants used one of three movement methods: VR-treadmill, VR-touchpad, or VR-teleportation. In the VR-treadmill condition, participants walked on the ROVR treadmill to move in VR. The treadmill consists of a dish-shaped platform with slippery surface, a waist-height containment frame, and a pair of low friction shoes (see Fig. 1). The treadmill detects the sound made by moving feet and converts this audio signal into forward motion in VR. In both VR-touchpad and VR-teleportation conditions, participants used a handheld HTC Vive controller (see Fig. 1) for translational movements. In the VR-touchpad condition, when participants touched the touchpad, participants moved through space at the speed of 1.4 m/s with continuous optic flow. Participants navigated at a slightly faster speed than the simulated avatars (1.3 m/s), so that they could overtake simulated avatars when necessary. In the VR-teleportation condition, participants moved through space with intermittent optic flow, by pressing a button on the controller, aiming toward an intended location, and releasing the button to be teleported to that location. Notably, each teleportation was not the same distance (< 5 m). We let participants actively control locomotion (in four conditions), as efferent motor commands on locomotion plays an important role in spatial learning (Chrastil and Warren 2012). However, we constrained the teleportation points to the central lane of main hallways (about 1–4 m wide depending on the width of hallways). Participants were not allowed to walk in a zig-zag manner or walk backwards, although a small curve was natural and inevitable, as navigators had to avoid other pedestrians during walking. For all three VR conditions, physical head rotations in the real world were directly mapped onto rotations in VR.

#### Perceived distance judgment

**Fig. 4:**
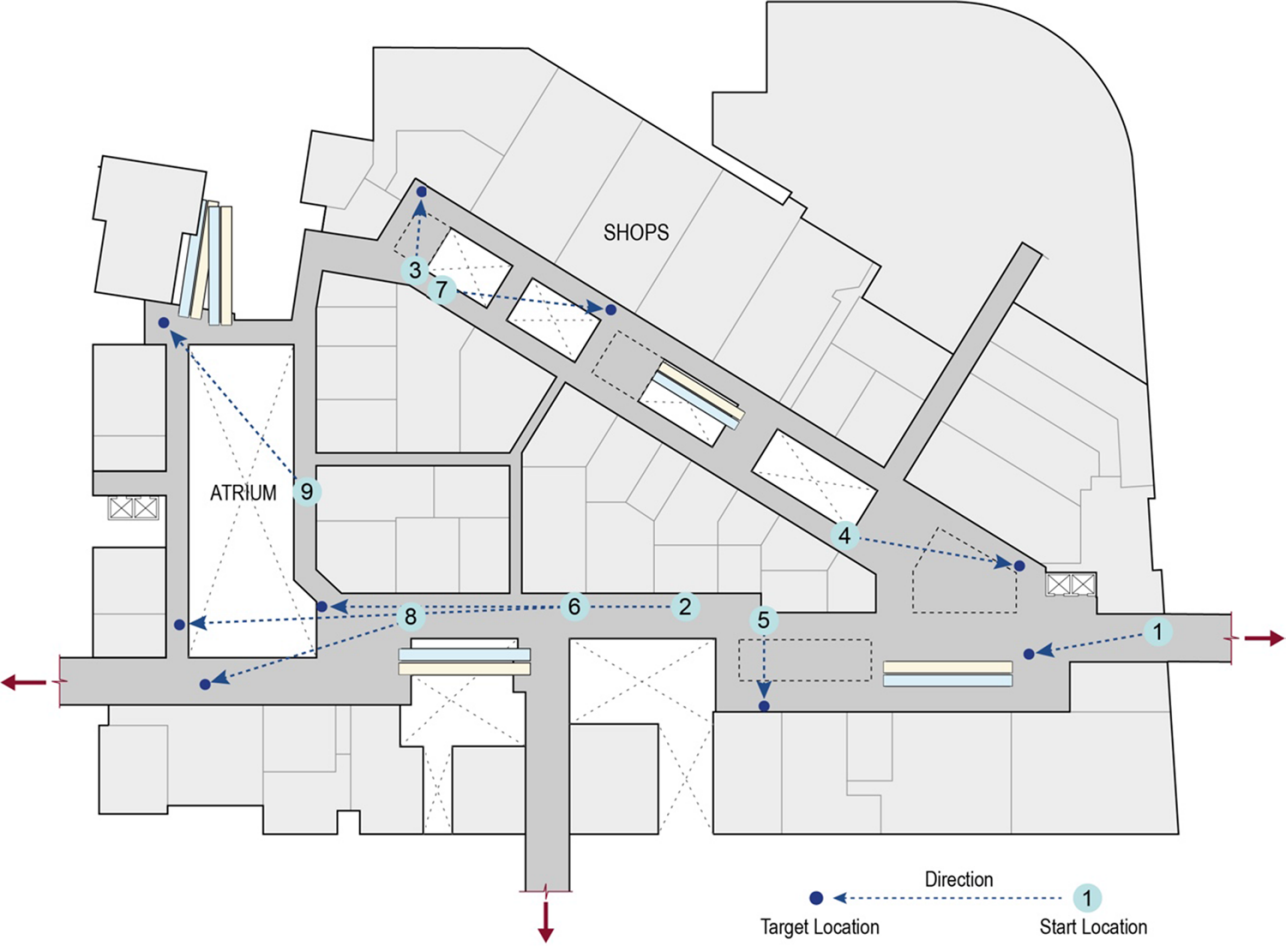
Nine start locations and targets for perceived distance estimates (distances ranging from 8 m to 49 m)

Participants completed nine trials of perceived distance estimates in all conditions (see Fig. 4). In three VR conditions, we used a red cylinder (3 m tall) located on the floor as the target; in the real world, we used a red board held at the height of approximately 3 m as the target. This ensured good visibility to the target in all conditions even though there were obstacles and crowds. We chose nine different start locations across all walkable areas, in order to achieve representative distance ranges and environmental features for distance estimates in a commercial indoor space.

#### Traversed distance judgment

**Fig. 5:**
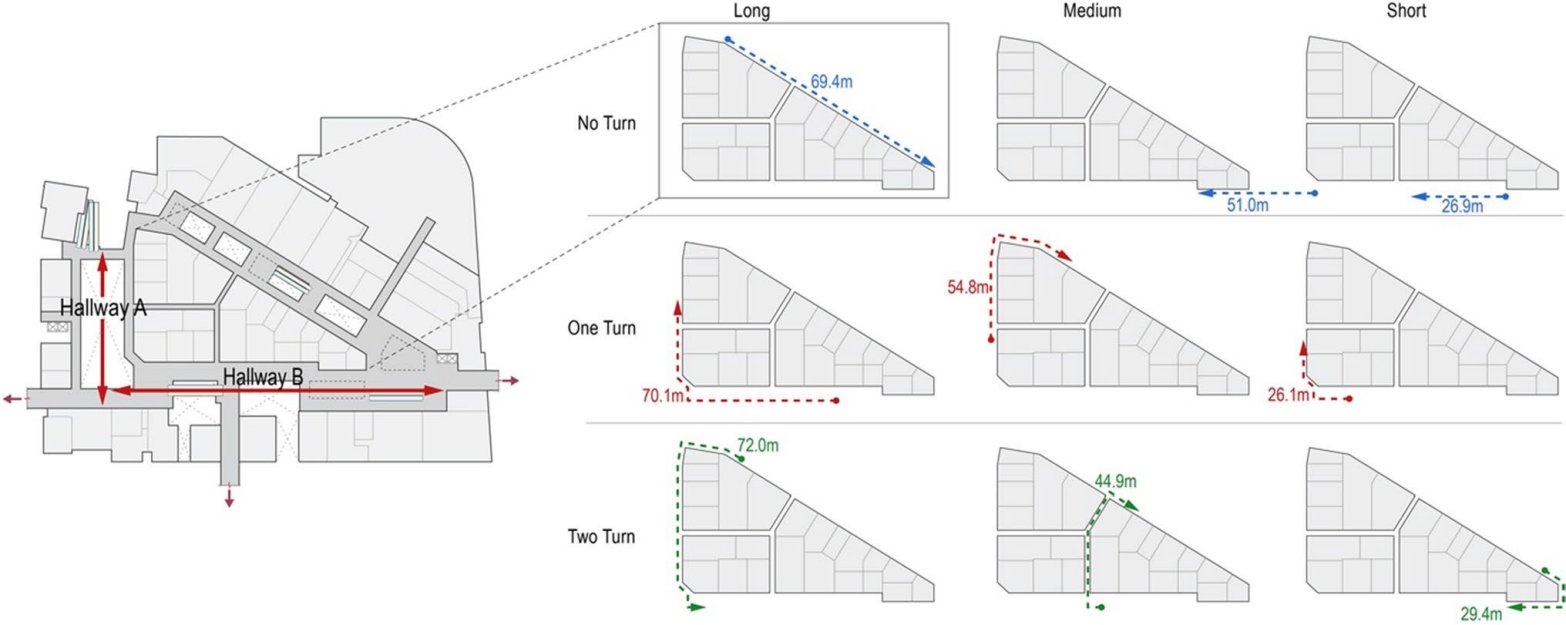
Nine routes for traversed distance estimates (distances ranging from about 26 m to 72 m). Hallways A and B represent the two perpendicular main hallways of the triangle-shaped floor

Participants completed nine trials of traversed distance estimates in all conditions (see Fig. 5). Participants walked a predetermined route and immediately provided a distance estimate; this was repeated for nine trials. We devised nine routes across all walkable areas, varied by the number of turns (0 to 2) and route length (short, medium, and long). Notably, this study was not designed to examine the effect of a specific number of turns or route length on distance perception, so both the number of turns and route length were treated as random effects in data analysis.

#### Environmental distance judgment

**Fig. 6:**
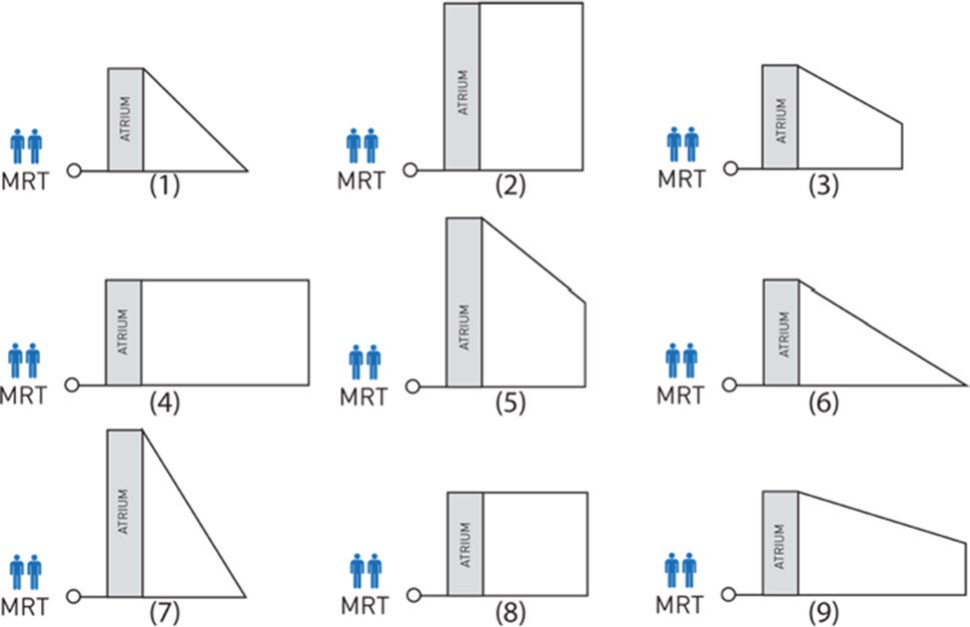
Nine options presented for the map-selection task. MRT refers to a public transit station, and the atrium beside MRT is another landmark of the environment used for orientation of the layout

For the judgement of environmental distance, participants performed a sketch map task followed by a map-selection task. In the map-selection task, participants were asked to choose one out of nine schematized layouts (see Fig. 6), that they thought most accurately represents the spatial configuration of the building. All layouts were simplified in a way that architectural details (columns, short hallways, etc.) were omitted. In this manner, we can ensure that participants chose the correct layout based on the learning of overall configurational metric knowledge (i.e., environmental distance) rather than irrelevant details. Among the nine options, both layouts 6 and 9 were correct answers with respect to environmental distance ratios and relative directions among the main hallways.

### Procedure

**Fig. 7:**
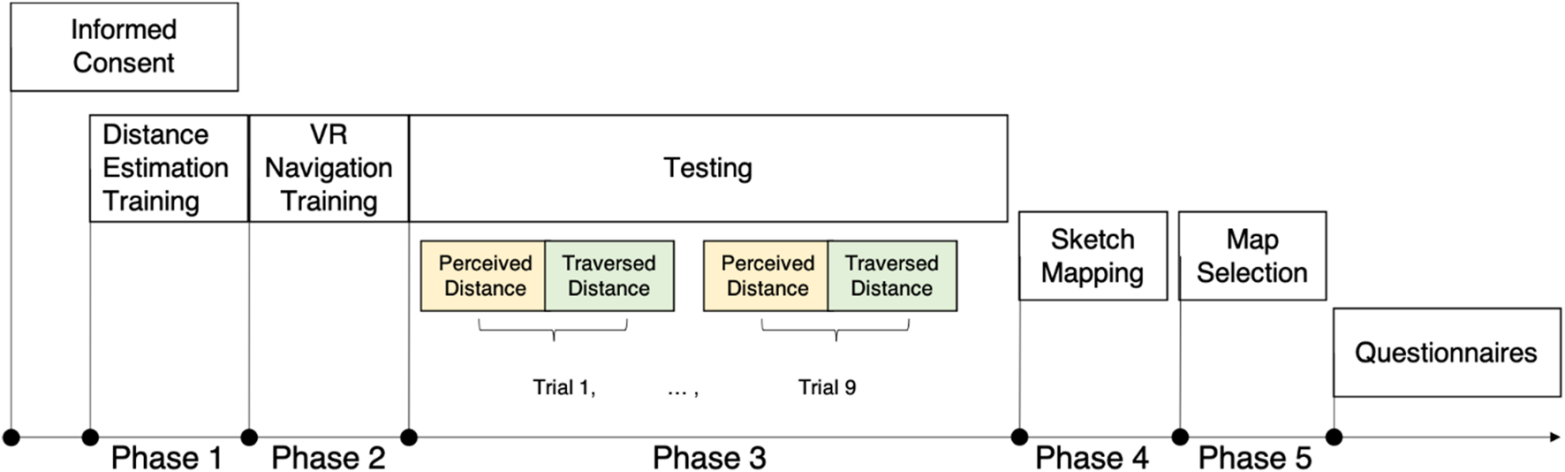
Experimental procedure

There were five phases in this experiment, as illustrated in Fig. 7.

Phase 1: Distance estimation training. The experimenter first introduced the tasks of perceived and traversed distance estimations. All participants reported that they understood the instructions. In the real-world condition, participants completed four practice trials for perceived distance (1 m, 2 m, 5 m, and 10 m) and one practice trial for traversed distance (22 m with one turn). In three VR conditions, participants had to first complete the real-world training followed by the VR training (i.e., four trials of perceived distance and one trial of traversed distance in VR). Participants were given corrective feedback about estimated distance after each practice trial
Phase 2: VR navigation training. In three VR conditions, participants had to learn how to move through VR using either treadmill, touchpad, or teleportation. The experimenter first demonstrated and explained how to use the movement method. Participants then practiced to move around in VR using one of these three movement methods. Participants had the opportunity to ask questions regarding the movement method and the tasks. All questions were answered, and all participants could easily navigate in VR before the distance estimation tasks
Phase 3: Testing perceived and traversed distance estimates. At the beginning of each trial, participants stood at a start location and were asked to verbally estimate perceived distance between their current location and a visible target. Participants were then instructed to follow the experimenter along a route toward a destination. After arriving at the destination, participants were asked to verbally report traversed distance of the route. During walking, participants were not allowed to count steps but were encouraged to pay attention to surrounding environments. When participants passed by the MRT (i.e., a public transit station), the experimenter reminded them that the MRT and the atrium beside it would serve as the landmarks in future tasks, but did not inform participants about the sketch map task and the map-selection task. At the end of each trial, participants were asked to point back to the start location. The pointing data were not analyzed here, as due to technical issue pointing data in the real-world condition were partly missing. After the nine perceived and traversed distance estimation trials, participants were guided to a room where they could not see the walked environment. In the VR conditions, participants simply removed the HMD
Phase 4: Sketch-mapping task. The experimenter asked participants to draw a sketch map on a provided single A4 sheet of paper of the learned floor with important landmarks labeled (e.g., the MRT)
Phase 5: Map-selection task. The experimenter presented a sheet of paper with nine possible layouts of the environment. The MRT and the atrium marks were explained. This instruction ensured that participants could correctly align their mental representation of the learned environment with respect to the layouts on the paper. Participants then chose a layout that best represented the learned environment

At the end of the experiment, participants in three VR conditions completed two questionnaires on HMD side effects and usability (not included in the analyses in this paper).

### Design and analysis

We collected a total of 1440 distance estimations (720 perceived and 720 traversed). Distance estimations were treated as outliers if their *Z*-score values were greater than 3 or less than -3. We detected 12 perceived and 15 traversed distance estimate outliers, all of which had *Z*-scores greater than 3. We decided to remove these outliers rather than correcting them using the mean mainly due to the fact that these outliers were erroneous. 20 out of these 27 outliers were caused by four participants. These participants performed worse on distance estimations for certain reasons such as they might forget the start location. For example, one participant estimated a 55 m (actual distance) traversed distance as 160 m. Some outliers might be due to that verbally reported distances were incorrectly recorded. Nevertheless, removing these outliers did not affect the overall results, and the outliers were almost evenly distributed across three conditions (real-world walking: 5 perceived and 4 traversed, VR-touchpad: 3 perceived and 3 traversed, and VR-teleportation: 4 perceived and 8 traversed).

For both perceived and traversed distance estimation tasks, the dependent variable was the distance ratio, computed as the ratio of estimated distance to actual distance. We used linear mixed-effects models based on the nlme package (Pinheiro et al. 2018) in R. Three separate linear mixed-effects models were analyzed to compare distance estimates among different movement methods: (1) real-world walking versus VR-treadmill, (2) VR-treadmill versus VR-touchpad, and (3) VR-touchpad versus VR-teleportation. We entered movement method as a fixed effect and participants and trial number as two separate random effects for the three models. This method does not require aggregating data from separate trials, while accounting for possible differences between the difficulty of each trial, as well as for different base-line performance of each participant. In order to investigate the impacts of independent variables on both the accuracy and precision of traversed distance estimates, we compared both the means and variances among different movement methods. Three separate likelihood ratio tests were conducted to compare two mixed-effects models (homogeneous variance model versus heterogeneous variance model).

**Fig. 8:**
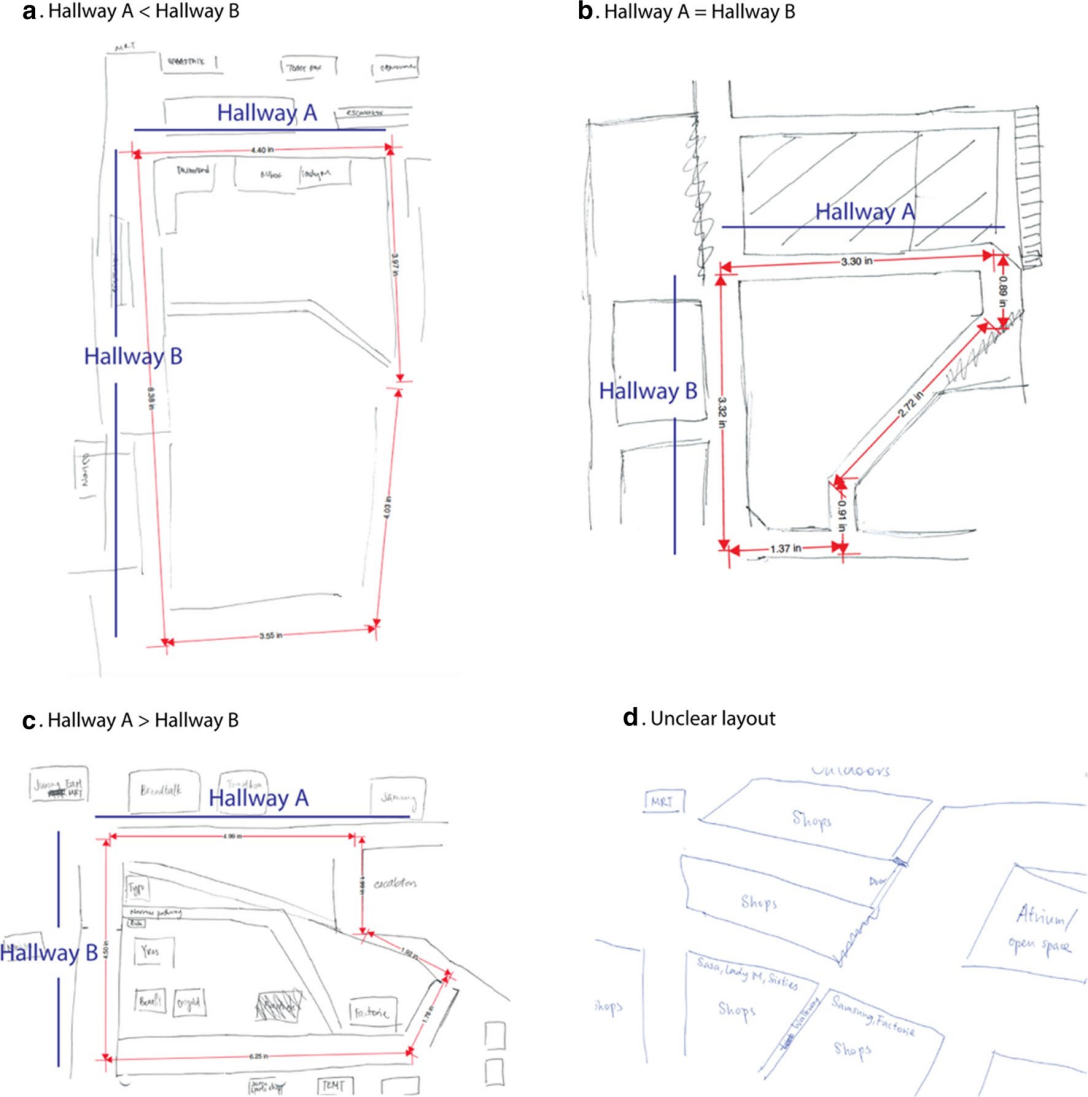
Four examples of sketch map drawings: correct environmental distance ratio (**a**) and incorrect environmental distance ratio (**b**, **c**, and **d**). Red lines represent the central lines of main hallways. Two blue lines indicate the two main hallways A and B

For the map-selection task, the dependent variable was the correctness of the chosen layout (two levels: correct vs. incorrect). Layouts 6 and 9 were correct with respect to environmental distance ratio and relative directions, and the others were incorrect. For the sketch map task, the dependent variable was the correctness of the sketch map in terms of environmental distance ratio (two levels: correct vs. incorrect). Three naive raters independently rated the correctness of all sketch maps. They were instructed to first draw central lines of main hallways based on the sketch maps and to then measure their distances (see Fig. 7). The experimenter later calculated the distance ratio between the two main hallways A and B (indicated by blue lines in Fig. 8). Inter-rater agreement was assessed using Fleiss’ *κ*, which indicated substantial agreement among the three raters for the classification of the distance ratio (*κ* = .62, *z* = 16.1, *p* < .001).

The actual environmental distance ratio between hallways A and B is about 0.6 (see Fig. 5). Even if participants successfully learned the actual environmental distance ratio, it was difficult to draw exactly accurate environmental distance ratio of 0.6, as hand-drawn sketch maps were likely to be affected by participants’ drawing abilities. Therefore, we used a tolerance of ± 0.2 (i.e., (1 − 0.6)/2) to categorize all environmental distance ratios into three levels: *A* < *B* (i.e., 0.4 ≤ *A*/*B* < 0.8), *A* ≈ *B* (i.e., 0.8 ≤ *A*/*B* ≤ 1.2), and *A *> *B* (*A*/*B* > 1.2). For example, if the environmental distance ratio between hallways *A* and *B* was smaller than 0.8 and greater than 0.4, we categorized the sketch map as correct environmental distance ratio. Otherwise, we categorized the sketch map as incorrect environmental distance ratio. For both the map-selection task and the sketch map task, we used Chi-squared tests of independence to assess whether the frequencies of choosing or drawing correct environmental distance ratio were independent of visual display, proprioception, and optic flow. The preceding analyses addressed hypotheses H1, H2, to H3. Here, we did not adopt bidimensional regression (Tobler 1994) as the sketch map analysis technique mainly due to that, in this study 23 participants drew unclear layouts (see Fig. 8d), making it impossible to implement quantitative bidimensional regression to assess the resemblance between two configurations. Thus, we only focused on environmental distances between two main hallways (i.e., two main directions). In addition, in the present study environmental distance was assessed by both the map-selection task and the sketch map task, so an ideal approach was to encode the dependent variables of both tasks in the same manner such as correct versus incorrect.

For H4, we conducted a series of t tests to compare the estimated distance ratio against the value of 1 (i.e., estimated distance equals actual distance) for each movement method. For H5, in order to study the relationship between actual distance (*X*) and estimated distance (*Y*) based on Stevens’ power law, we conducted nonlinear least-squares estimates of the exponent *n* and used actual distance to fit estimated distance.

For H6, we aimed to study whether participants who produced correct environmental distance ratios were more accurate in their perceived and traversed distance judgements. We first assessed the internal consistency between the two measures of environmental distance (Cronbach’s *α* = 0.688), which indicated reasonable agreement between the two measures (i.e., the correctness of the sketch map and the correctness of the map selection). We found that out of 24 participants who drew correct sketch maps, 8 failed to choose correct floor layouts. In total, 16 out of 80 participants were correct in both the sketch map task and the map-selection task. These 16 participants were subsequently categorized as the group of correct environmental distance ratio, and the other 64 participants were categorized as the group of incorrect environmental distance ratio. Here, we had unequally sized groups due to that 64 participants out of 80 did not learn accurate environmental distance. Unequal group sizes can lead to unequal variances between groups, which affects the homogeneity of variance assumption in tests like ANOVA. However, mixed-effects model is considered robust to moderate difference in group size by explicitly modeling heterogeneity at the individual level (Pinheiro et al. 2018). Thus, we compared perceived and traversed distance estimates between these two groups using mixed-effects models.

## Results

### Visual display

**Fig. 9:**
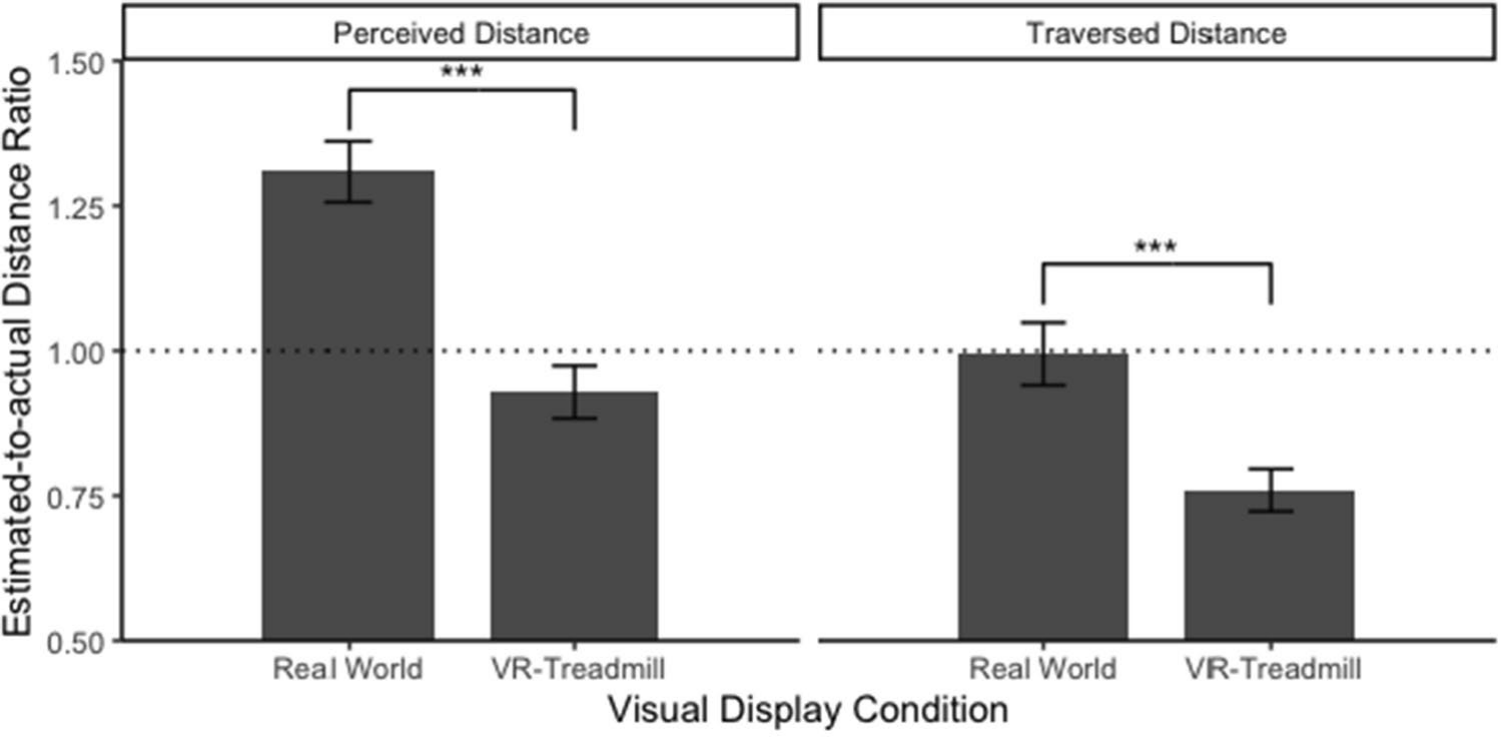
Average perceived and traversed distance ratio between the real world and the VR-treadmill conditions. The dashed line indicates correct distance estimates (response distance = actual distance)

The linear mixed models revealed a significant effect of visual display on perceived distance estimates, *b* = − 0.273, SE_*b*_ = 0.051, *t*(38) = − 5.403, *p *< .001, marginal *R*^2^ = 0.274. Participants in the real world (*M* = 1.309, SD = 0.353) tended to overestimate perceived distances compared to participants in the VR-treadmill condition (*M* = 0.929, SD = 0.309; see Fig. 9). The linear mixed models also revealed a significant effect of visual display on traversed distance estimates, *b* = − 0.167, SE_*b*_ = 0.043, *t*(38) = − 3.859, *p* < .001, marginal *R*^2^ = 0.154. Participants in the VR-treadmill condition (*M* = 0.760, SD = 0.249) were more likely to underestimate traversed distance compared to participants in the real world (*M* = 0.995, SD = 0.363; see Fig. 9). The likelihood ratio test indicated heterogeneous variance between the two conditions, likelihood ratio = 14.376, *p* < .001. Specifically, participants in the real-world condition (*σ*^2^ = 0.132) had larger variance of traversed distance compared to participants in the VR-treadmill condition (*σ*^2^ = 0.062).

The Chi-squared test of independence for environmental distance (measured by the sketch map task and the map-selection task) was not significant for sketch map: *χ*^*2*^(1) = 2.976, *p* = 0.085, Cramér’s *V* = 0.27, and map selection: *χ*^*2*^(1) = 0.456, *p* = 0.500, Cramér’s *V* = 0.107. Descriptive statistics for two environmental distance tasks are presented in Table 2. Together, these results demonstrated that visual display impacted the judgments of both perceived and traversed distance but not environmental distance. Notably, there might be a trend toward an effect of visual display on sketch map drawing given the medium effect size (i.e., Cramér’s *V* = 0.27). Nevertheless, as the effect of visual display on environmental distance estimates was not significant, we can conclude that H1 was validated.

**Table 2:** Frequency of drawing and choosing correct environmental distance in the sketch map task and the map-selection task for the real world and VR-treadmill conditions

Tasks	Correctness of environmental distance	Real-world walking	VR-Treadmill
Sketch map	Correct	9	3
	Incorrect	11	17
Map selection	Correct	8	5
	Incorrect	12	15

### Proprioception

**Fig. 10:**
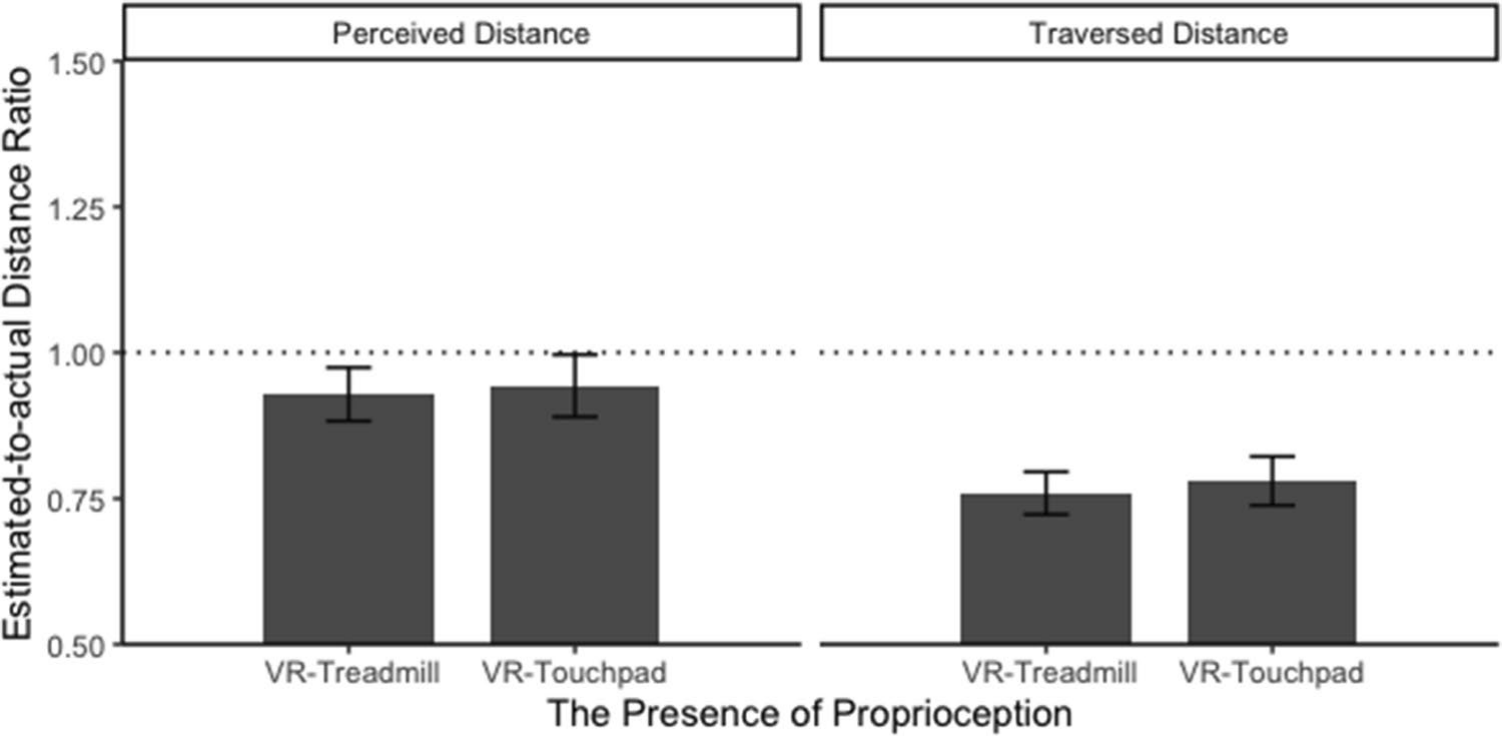
Average perceived and traversed distance ratio between the VR-treadmill and VR-touchpad conditions. The dashed line indicates correct distance estimates (response distance = actual distance)

The linear mixed models revealed no effects of proprioception on perceived and traversed distance estimates, perceived distance: *b* = 0.015, SE_*b*_ = 0.052, *t*(38) = 0.290, *p* = .773, marginal *R*^2^ = 0.001; traversed distance: *b* = 0.018, SE_*b*_ = 0.036, *t*(38) = 0.494, *p* = .624, marginal *R*^2^ = 0.002. Perceived distance estimates between the VR-treadmill (*M* = 0.929, SD = 0.309) and VR-touchpad (*M* = 0.943, SD = 0.357) conditions were similar (see Fig. 10). Traversed distance estimates between the VR-treadmill (*M* = 0.760, SD = 0.249) and VR-touchpad (*M* = 0.780, SD = 0.283) conditions were also similar (see Fig. 10). The likelihood ratio test indicated homogeneity of variance of traversed distance estimates between the VR-treadmill and the VR-touchpad conditions, likelihood ratio = 1.351, *p* = .245.

In order to validate the no-effect hypotheses H2.1 and H2.2, we repeated the above analyses in the Bayesian framework, by implementing equivalent models in the brms *R* package (Buerkner 2017) which is based on Stan (Carpenter et al. 2017). We used a recommended generic weakly informative prior^1^ on the main fixed effect, and default brms package priors on other parameters, deriving Bayes Factors (BF) using the bayestestR *R* package (Makowski et al. 2019). For the effect of proprioception on perceived distance estimates, BF_10_ = 0.19 (i.e., BF_01_ = 1/0.19 = 5.26), meaning that there is five times as much evidence for the no-effect hypothesis as for its alternative. This indicates *substantial* evidence for our hypothesis H2.1 (Wetzels et al. 2011). For the effect of proprioception on traversed distance estimates, BF_10_ = 0.17 (i.e., BF_01_ = 5.88), meaning that there is almost 6 times as much evidence for the no-effect hypothesis as for its alternative. This indicates *substantial* evidence for our hypothesis H2.2.

No effect of proprioception on the sketch map task and the map-selection task was observed, sketch map: *χ*^*2*^(1) = 2.006, *p* = 0.157, Cramér’s *V* = 0.22, and map selection: *χ*^*2*^(1) < 0.001, *p* > 0.999, Cramér’s *V* < 0.001. Descriptive statistics for two environmental distance tasks are presented in Table 3. Together, these results suggested that proprioception did not impact the estimations of perceived distance, traversed distance, and environmental distance. These results partially supported H2 (H2.3 was not supported).

**Table 3:** Frequency of drawing or choosing correct environmental distance in the sketch map task and the map-selection task for the VR-treadmill and VR-touchpad conditions

Tasks	Correctness of environmental distance	VR-Treadmill	VR-Touchpad
Sketch map	Correct	3	8
	Incorrect	17	12
Map selection	Correct	5	6
	Incorrect	15	14

### Continuity of optic flow

**Fig. 11:**
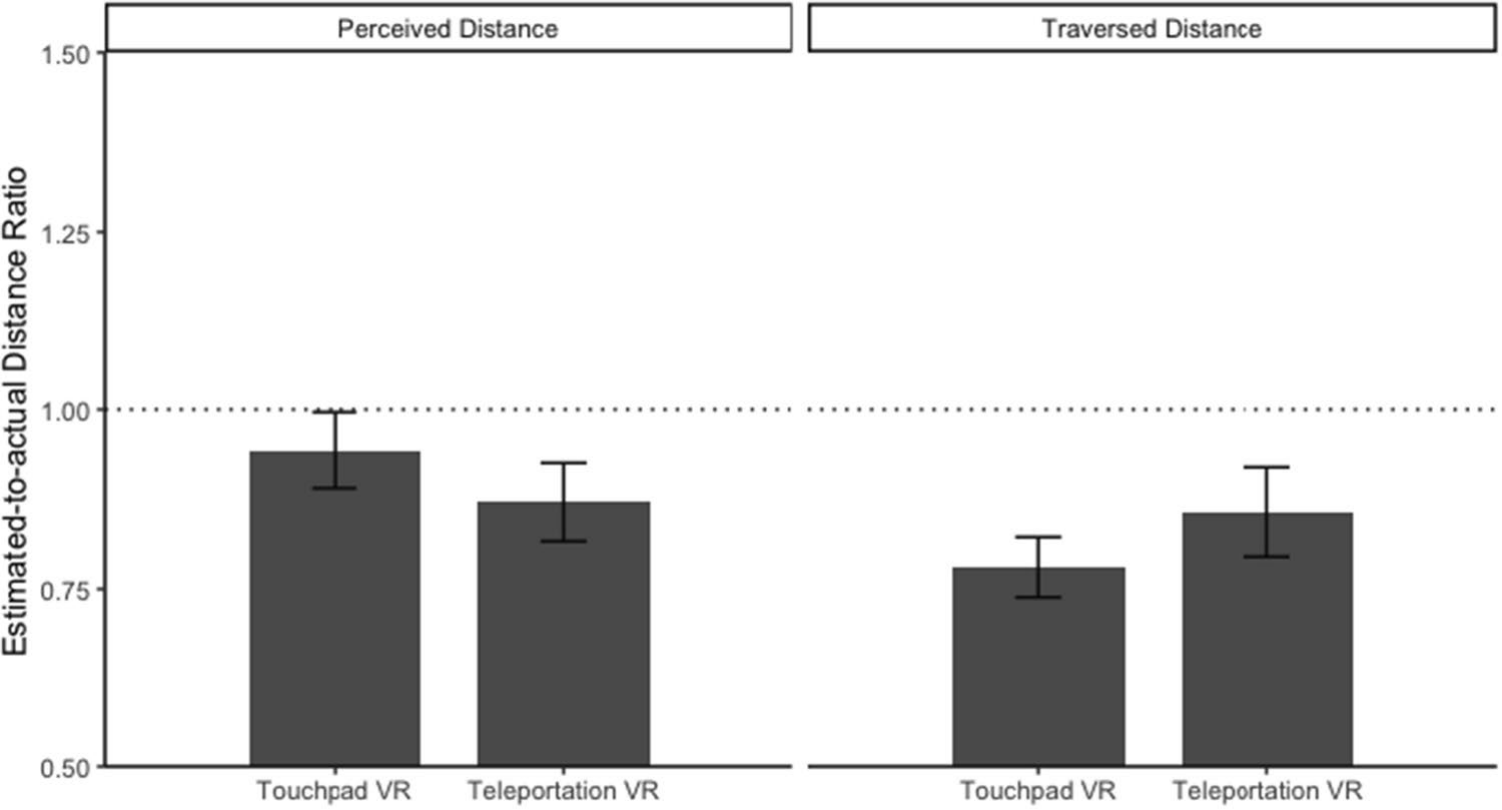
Average perceived and traversed distance ratio between the VR-touchpad and the VR-teleportation conditions. The dashed line indicates correct distance estimates (response distance = actual distance)

The linear mixed models revealed no effect of the continuity of optic flow on perceived and traversed distance estimates, perceived distance: *b* = − 0.050, SE_*b*_ = 0.060, *t*(38) = 0.821, *p* = .412, marginal *R*^2^ = 0.009; traversed distance: *b* = 0.065, SE_*b*_ = 0.060, *t*(38) = 1.085, *p* = .285, marginal *R*^2^ = 0.017. Perceived distance estimates between the VR-touchpad (*M* = 0.943, SD = 0.357) and the VR-teleportation (*M* = 0.871, SD = 0.366) and conditions were similar (see Fig. 11). Traversed distance estimates between the VR-touchpad (*M* = 0.780, SD = 0.283) and the VR-teleportation (*M* = 0.857, SD = 0.414) conditions were also similar (see Fig. 11). The likelihood ratio test indicated heterogeneous variance of traversed distance estimates between the VR-touchpad and the VR-teleportation conditions, likelihood ratio = 3.953, *p* = .047. Participants in the VR-teleportation condition had larger variance (*σ*^2^ = 0.171) compared to participants in the VR-touchpad condition (*σ*^2^ = 0.080).

In order to validate the no-effect hypotheses H3.1 and H3.2, we repeated the above analyses in the Bayesian framework. For the effect of the continuity of optic flow on perceived distance estimates, BF_10_ = 0.23 (i.e., BF_01_ = 4.35), meaning that there is over four times as much evidence for the no-effect hypothesis as for its alternative. This indicates *substantial* evidence for our hypothesis H3.1. For the effect of the continuity of optic flow on traversed distance estimates, BF_10_ = 0.30 (i.e., BF_01_ = 3.33), meaning that there is 3 times as much evidence for the no-effect hypothesis as for its alternative. This indicates *substantial* evidence for our hypothesis H3.2.

No effect of the continuity of optic flow on environmental distance was observed, sketch map: *χ*^*2*^(1) = 1.071, *p* = 0.301, Cramér’s *V* = 0.16, and map selection: *χ*^*2*^(1) < 0.001, *p* > 0.999, Cramér’s *V* < 0.001. Descriptive statistics for two environmental distance tasks are presented in Table 4. These results suggest that the continuity of optic flow did not impact the estimations of environmental distances. These results partially supported H3 (H3.3 was not supported).

**Table 4:** Frequency of drawing or choosing correct environmental distance in the sketch map task and the map-selection task for the VR-touchpad and VR-teleportation conditions

Tasks	Correctness of environmental distance	VR-touchpad	VR-Teleportation
Sketch map	Correct	8	4
	Incorrect	12	16
Map selection	Correct	6	5
	Incorrect	14	15

### The comparison between estimated distance and actual distance

A series of t tests (i.e., estimated distance ratio against the value of 1) revealed that participants significantly overestimated perceived distance in the real world and significantly underestimated perceived distance in each of the three VR conditions: real-world walking: *t*(174) = 11.583, *p* < .001, Cohen’s *d* = 0.88; VR-treadmill: *t*(179) = -3.096, *p* = .002, Cohen’s *d* = 0.23; touchpad VR: *t*(176) = -2.11, *p* = .036, Cohen’s *d* = 0.16; VR-teleportation: *t*(175) = -4.667, *p* = .036, Cohen’s *d* = 0.35. Those t tests also revealed that participants underestimated traversed distance in the three VR conditions: VR-treadmill: *t*(179) = -12.979, *p* < .001, Cohen’s *d* = 0.97; VR-touchpad: *t*(176) = -10.329, *p* < .001, Cohen’s *d* = 0.78; VR-teleportation: *t*(171) = -4.520, *p* < .001, Cohen’s *d* = 0.35. Interestingly, there was no significance for traversed distance estimates in the real world compared to actual distance, *t*(175) = -0.200, *p* = .841, Cohen’s *d* = 0.02. Together, these results suggested that participants tended to underestimate both perceived and traversed distances in VR but overestimated only perceived distance in the real world (see Figs. 9, 10, and 11). Accordingly, H4 was supported. Notably, we found that traversed distance based on verbal report in the real world was accurate compared to actual distance.

### The function between actual distance and estimated distance

**Fig. 12:**
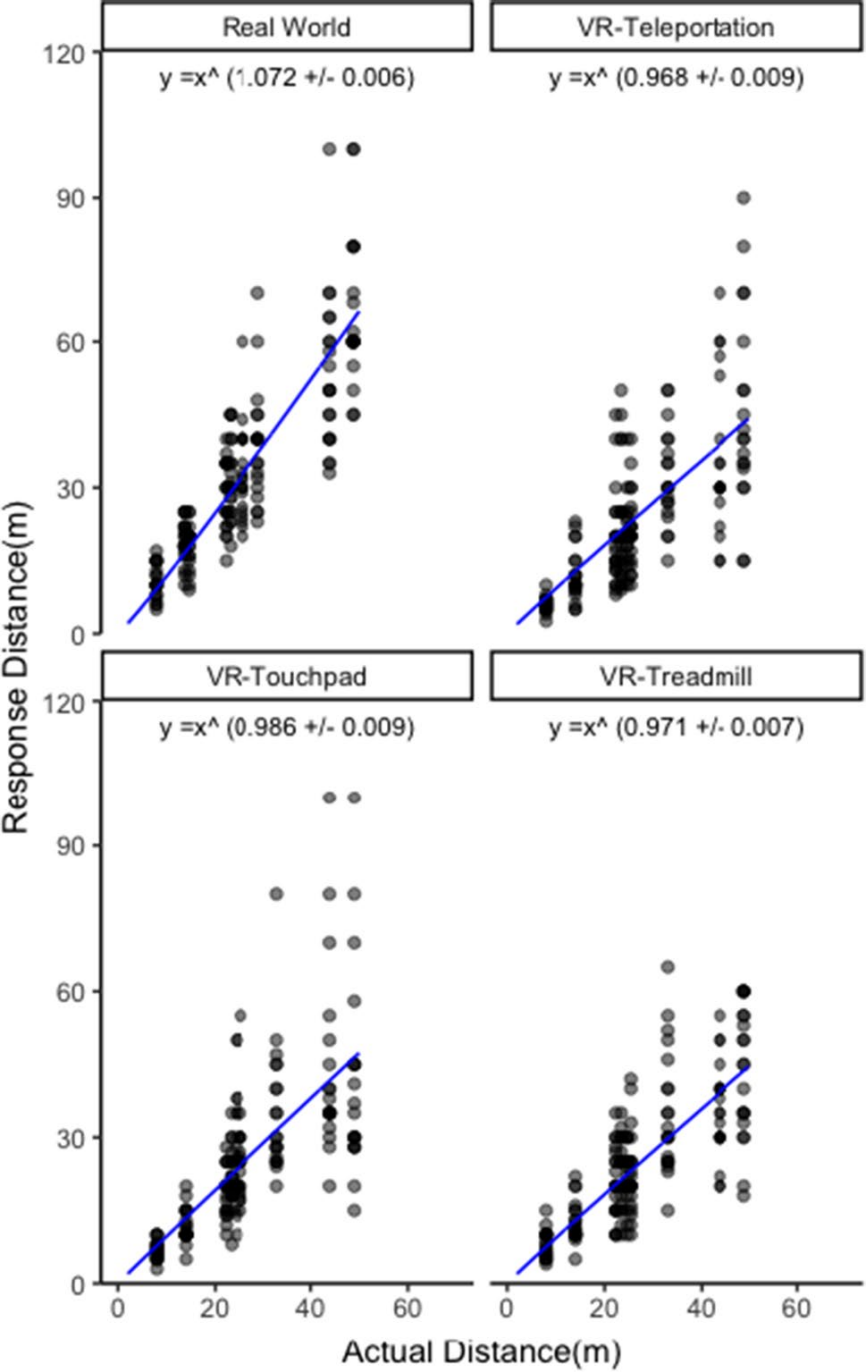
The relationship between actual distance and the estimation of perceived distance based on Stevens’ power law

The nonlinear least-squares estimate model revealed a positive acceleration of the power function between actual distance and estimated distance for perceived distance in the real world, exponent *n* = 1.072, *t*(174) = 189.7, *p* < .001, *R*^2^ = 0.773. Thus, H5 was supported. Notably, there were three negative accelerations of the power function in VR; treadmill VR: exponent *n* = 0.971, *t*(179) = 142.9, *p* < .001, *R*^2^ = 0.592; touchpad VR: exponent *n* = 0.986, *t*(176) = 110.2, *p* < .001, *R*^2^ = 0.487; teleportation VR: exponent *n* = 0.968, *t*(175) = 108.3, *p* < .001, *R*^2^ = 0.510 (see Fig. 12).

### The relationship between environmental distance and perceived (or traversed) distance

**Fig. 13:**
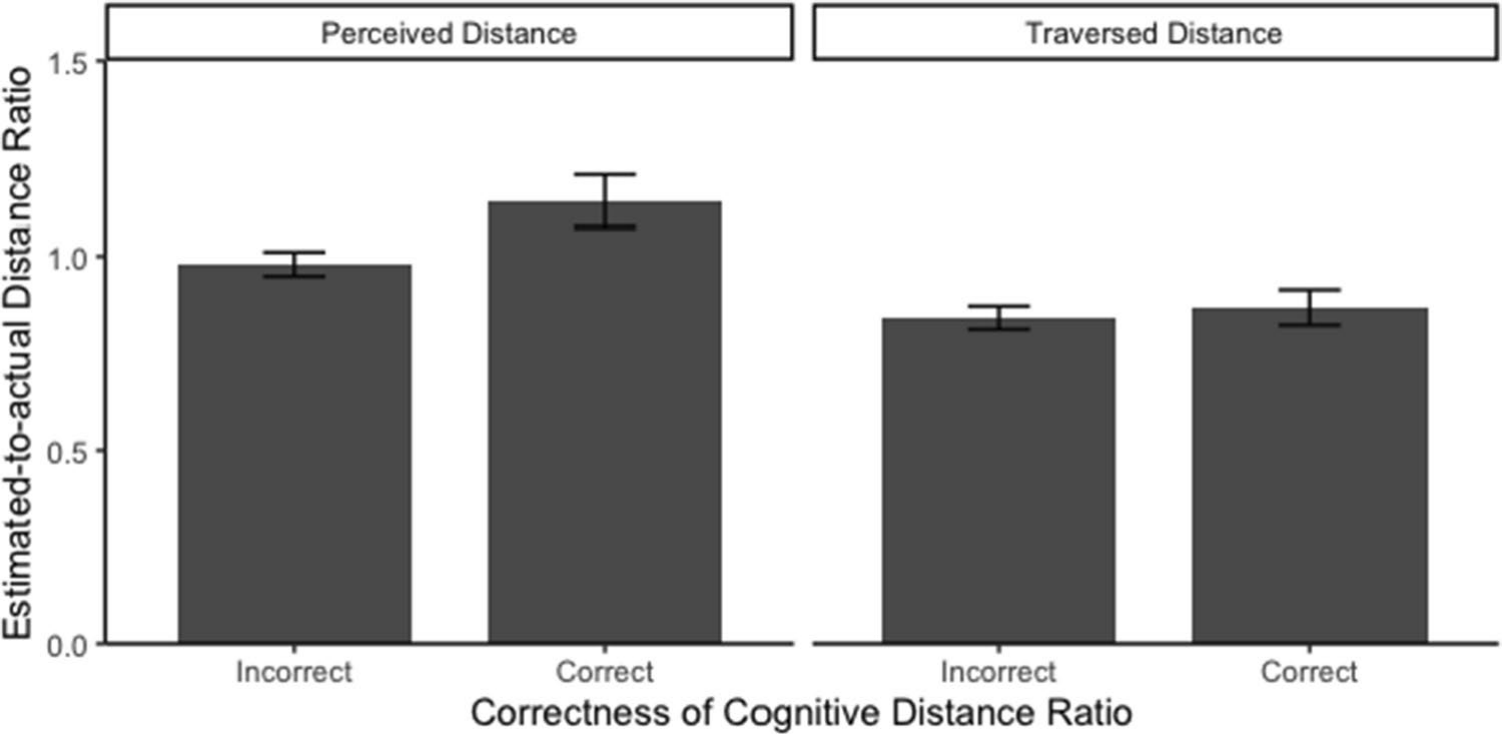
Average perceived and traversed distance ratio between two groups of participants (incorrect environmental distance ratio versus correct environmental distance ratio)

The linear mixed models revealed no effect of the correctness of environmental distance on the estimations of perceived or traversed distances, perceived distance: *b* = 0.155, SE_*b*_ = 0.083, *t*(78) = 1.863, *p* = .066, marginal *R*^2^ = 0.025; traversed distance: *b* = 0.017, SE_*b*_ = 0.069, *t*(78) = 0.246, *p* = 0.806, marginal *R*^2^ < .001. The results showed that participants who produced more accurate environmental distance judgements in the sketch map task and the map-selection task were not necessarily more accurate in estimating perceived distances (*M* = 1.139, SD = 0.406) and traversed distances (*M* = 0.868, SD = 0.277), compared to participants who produced incorrect environmental distance estimates (perceived distances: *M* = 0.980, SD = 0.375; traversed distances: *M* = 0.842, SD = 0.360; see Fig. 13). In order to validate the no-effect hypothesis H6, we repeated the above analyses in the Bayesian framework. For the effect of the correctness of environmental distance on the perceived distance estimates, BF_10_ = 0.82 (i.e., BF_01_ = 1.22), meaning that there is only 1.22 times as much evidence for the no-effect hypothesis as for its alternative and the result is inconclusive (i.e., there was no substantial support for the null hypothesis, and no substantial support for its alternative). For the effect of the correctness of environmental distance on the traversed distance estimates, BF_10_ = 0.22 (i.e., BF_01_ = 4.54), meaning that there is over 4 times as much evidence for the no-effect hypothesis as for its alternative. This indicates substantial evidence for this part of our hypothesis H6. Thus, H6 was partially supported (the result of the perceived distance estimates analysis was inconclusive).

## Discussion

The primary goal of this study was to investigate the role of visual display, proprioception, and the continuity of optic flow on perceived, traversed, and environmental distance estimation in a large-scale building. Participants were tested in four movement methods including real-world walking, VR-treadmill, VR-touchpad, and VR-teleportation. These movement methods provide different sensory information. Real-world walking provides natural visual input and proprioception. VR-treadmill provides virtual visual input with continuous optic flow and proprioception. VR-touchpad allows for continuous optic flow but did not provide proprioceptive input. Finally, VR-teleportation only allows for intermittent optic flow. We tested how different sensory information impacts perceived and traversed distance perception (measured by verbal report) and environmental distance learning (measured by a map-selection task and a sketch-mapping task) in a large-scale public building.

### Visual display and distance estimates

The results revealed that visual display affected the judgments of both perceived and traversed distances. Specifically, participants in the real-world walking condition overestimated both perceived and traversed distance compared to participants in the VR-treadmill condition. In this study, we used the new-generation HMD (HTC Vive) with highly rendered virtual environments including textures, ambient lighting, shadows, and simulated avatars, but certain visual depth clues such as reflections were still lacking. Although previous literature based on old HMDs has found that both field of view and resolution did not affect distance perception in VR (for a review, see Creem-Regehr et al. 2005, 2015b; Ijsselsteijn et al. 2001; Knapp and Loomis 2004; Polys et al. 2007), it is still important to examine the effect of the latest HMDs such as pimax 8 K on distance perception. Largely increased image quality (e.g., dual naive 4 K displays) might reduce screen door effect (i.e., VR users feel like looking through a screen door due to the space between pixels) and might mitigate the compression of perceived distance in VR.

In regards to traversed distance, we found that participants tended to underestimate traversed distance in VR, which is consistent with the finding of Witmer and Kline (1998). They explained that the compression of traversed distance in VR was likely due to the dominance of visual cues in VR (usually underestimated) over nonvisual distance cues in traversed distance estimation. They also found that participants provided with compensatory nonvisual distance cues (i.e., a beep sounded once for every 10 feet of movement) better estimated the length of traversed distances. In the present study, we found that traversed distance estimates were accurate compared to actual distance. One possible explanation was that participants in the real world were more likely to count steps compared to participants in three VR conditions. Although participants were told not to count steps while walking, participants in the real world might still use this strategy. This issue should be addressed in a future study.

We found no effect of visual display on the judgements of environmental distance, which was measured as the ratio between hallways A and B. The lack of visual display effect on the learning of environmental distance was likely due to a floor effect. In order to mimic the real-world way finding behaviors in a shopping mall, the experimenter did not ask participants to intentionally learn the building layout and did not inform them about the sketch-map and map-selection tasks beforehand. Partly due to this control, most participants (both in the real world and VR conditions) failed to build accurate cognitive maps, evidenced by the fact that only 20% of participants (16 out of 80) reproduced the correct environmental distance ratio. We acknowledge that the measure chosen to assess environmental distance might also affect the visual display effect. Future studies should let participants learn the environment for a longer time and assess built spatial representation by comparing not only distance ratios but also landmarks and configurations.

Taken together, these finding indicate that, although perceived and traversed distance estimates between the real world and VR are different, spatial learning in a real environment and in VR are similar, which is consistent with previous literature on the validity of using VR as a testbed for spatial cognition research (Loomis et al. 1999a; Richardson et al. 1999; Ruddle et al. 1997). Notably, we observed a trend toward an effect of visual display on sketch map drawing given the medium effect size. This issue should also be addressed in a future work with a larger sample size.

### Proprioception and distance estimates

The results showed that proprioception did not affect perceived and traversed distance estimates. The lack of proprioception effect on perceived distance estimates is as expected, because no translations were involved during the judgements of perceived distances. Participants in three VR conditions used exactly the same visual display, stood at the same locations, and did not move during the estimation of perceived distances. On the other hand, this finding indicated that perceived distance estimates in the present study were consistent and reliable across three VR conditions.

The lack of proprioception effects on traversed distance estimates is consistent with some studies (Bremmer and Lappe 1999; Richardson et al. 1999; Waller and Greenauer 2007; Witmer and Kline 1998). As discussed above, the lack of proprioception effects﻿ on traversed distance was likely due to that participants primarily relied on visual cues for traversed distance estimates. However, this finding is inconsistent with the work of Campos et al. (2012), in which both proprioceptive and vestibular cues were found to contribute to travelled distance estimates during walking in a large free-walking space. On possible reason for this inconsistency was that in our present study traversed distance estimates involved turns (0 to 2), and proprioceptive cues might be less reliable for traversed distance estimation with turns in a large-scale space. Another possible reason was that the ROVE treadmill was not intuitive enough to walk on. The unnatural movement of shuffle-sliding one’s feet on the ROVE treadmill might have distracted participants from attending to traversed distance because of the physical effort. Together, our current findings suggest that proprioception is not a primary determinant of traversed distance estimate in a large-scale indoor environment.

We did not find the effect of proprioception (VR treadmill versus VR touchpad) on the learning of environmental distance. This finding is inconsistent with the finding of Ruddle et al. (2011). They found that participants who walked using a treadmill had built more accurate survey knowledge compared to participants who used a joystick for navigation. Similar to the discussion of visual display, the inconsistency was likely due to the floor effect (i.e., most participants failed to build correct environmental distance). According to Chrastil and Warren (2012), “it may require a sufficiently complex path or repeated exposure for idiothetic information to reveal its effect.” Future studies should design more complex environment than our current one and expose participants in the environment for a longer time, in order to study the effect of proprioception on traversed distance estimates.

### Optic flow and distance estimates

We found that intermittent optic flow (VR-teleportation) did not affect the overall accuracy of traversed distance estimations but impaired the precision of these estimations. Specifically, participants in the VR-teleportation condition (intermittent optic flow) had larger variance of traversed distance estimations compared to participants in the VR-touchpad condition (continuous optic flow). This finding is inconsistent with Witmer and Kline (1998)’s work, in which the continuity of optic flow did not affect traversed distance estimates in VR. The most important reason for this inconsistency was that Witmer and Kline (1998) did not compare the variances between the teleportation and joystick conditions. Another possible explanation is that in Witmer and Kline (1998)’s study participants were passively teleported by the experimenter, whereas in the present study participants actively teleported themselves. Thus, travel speed between our current study and the work of Witmer and Kline (1998) was likely to be different, and movement speed is a major factor affecting traversed distance estimates (Witmer and Kline 1998). Apart from experimental procedure, different materials between the two studies (e.g., floor layout, VR rendering, and HMDs) might also contribute to this inconsistence.

We did not find the effect of the continuity of optic flow (VR touchpad versus VR teleportation) on the learning of environmental distance. This finding is somewhat inconsistent with previous works that the teleportation interface impaired spatial awareness (Moghadam et al. 2020), spatial updating (Cherep et al. 2020), and spatial orientation (Bhandari et al. 2018). This inconsistency was also likely due to the floor effect such that most participants failed to build correct environmental distance.

Taken together, although teleportation is now a mainstream VR movement technique in VR applications and games (Boletsis 2017), our results indicated that teleportation may not be the best movement method for spatial cognition studies in VR due to the inconsistency in establishing mental representations of distances as evidenced by large variance in traversed distance estimates. In contrast, the VR-touchpad movement method requires a less complex VR setup than a treadmill and does not impact traversed distance estimates, making it a more viable movement method for spatial cognition research in VR. New VR walking platforms and novel interaction methods such as arm swinging (e.g., Wienrich et al. 2019) are making movement in VR much easier than ever before. Future research should examine the effects of these new methods on human spatial learning and mental representation.

### The comparison between estimated distance and actual distance

With respect to the comparison between estimated distances to actual distances, we found that participants tended to underestimate both perceived and traversed distances in VR compared to actual distances. These findings are somewhat consistent with previous literature (Creem-Regehr et al. 2015a; Kelly et al. 2017; Renner et al. 2013; Witmer and Kline 1998). In contrast to previous studies, we observed less underestimation of perceived distance in VR in this study (91% of actual distance compared to previous studies on average 74% of actual distance; see Renner et al. 2013 for comprehensive review). We see two possible reasons. First, we used a newer HMD in this study. Recent research with comparable HMDs (e.g., HTC Vive and Oculus Rift DK2) have found higher accuracy in distance estimates compared to older displays (Creem-Regehr et al. 2015b; Kelly et al. 2017). Newer HMDs are not only equipped with better displays providing higher pixel density but also are lighter with better ergonomic design. Notably, previous literature has found that mechanical aspects of HMDs such as mass and moments of inertia to certain extent caused distance underestimation in VR (Willemsen et al. 2004). The second reason was that we conducted distance estimation training beforehand. Previous literature had found that a short period of feedback training can improve distance judgements both in the real world (Gibson et al. 1955; Gibson and Bergman 1954) and in VR (Richardson and Waller 2005). Nevertheless, compared to perceived distance estimates in the real world, the judgements of perceived distance in VR were still largely underestimated.

We observed a positive acceleration of the power function (the exponent *n* = 1.072) between actual distance and estimated distance for perceived distance in the real world. In other words, as actual distance increases, people tend to increasingly overestimate distance. Even though the exponent is close to 1.00, this finding is important because it indicated a surge of distance estimate errors for further away targets. For example, based on the power function model (*n* = 1.072), the predicted distance estimation of a 20 m target is only about 25 m (distance estimate error = 5 m), but the prediction of a 200 m target is 293 m (distance estimate error = 93 m). This finding is consistent with previous works on psychophysics (Künnapas 1960; Luria et al. 1967; Teghtsoonian and Teghtsoonian 1969). However, the exponent observed in the present study (*n* = 1.072) was lower compared to previous studies (*n* ranges from 1.2 to 1.5). It was likely due to the distance estimation training discussed above. Another possible explanation is that the present study used different indoor environmental features compared to previous literature. For example, the building included a large atrium, and there were pedestrian crowds walking between the observer and the target. In contrast to the finding in the real world, we observed negative acceleration of the function between actual distance and estimated distance in VR. This finding indicates that perceived distance estimates in VR undergoes exponential decay—the farther the target is, the larger underestimation the estimated distance is. This issue should be addressed in a future study.

### The relationship between environmental distance and perceived (or traversed) distance

We found that the accuracy of learning the environmental distance of a large-scale built environment was not associated with the accuracy of traversed distance estimates in that environment. However, the association between environmental distance learning and the accuracy of perceived distance estimates was inconclusive. Together, we did not find solid evidence about any associations between the judgements of environmental distance and perceived (or traversed) distance. This finding is somewhat consistent with previous literature on representational flexibility in that navigators acquire allocentric and egocentric spatial knowledge in parallel (Brunyé et al. 2008; Iglói et al. 2009). Although all participants learned the environment from the same perspective, individual differences and different learning goals might determine that participants performed differently at egocentric and allocentric spatial knowledge (Meilinger et al. 2016; Pazzaglia and Taylor 2007). This finding was also likely due to the specific measure chosen to assess environmental distance. In both the map-selection task and the sketch-mapping task, we used the distance ratio between two main hallways to assess the learning of environmental distance (i.e., relative length). However, the absolute lengths of all hallways were unknown, as no scale was provided on the map. It was possible that participants who estimated accurate perceived and traversed distances were more accurate in estimating the absolute length of one of the main hallways. This issue should be addressed in a future study.

One implication of this result is that accurate learning of environmental distance in a large-scale environment requires not only distance perception of local places but also (probably more importantly) the integration of subsequently learned local places into a coherent global mental representation. Tying back to the continuous framework of spatial knowledge acquisition (Ishikawa and Montello 2006; Montello 1998), although most navigators begin to acquire metric configurational knowledge on the first exposure to a new large-scale environment, the initially learned metric knowledge is not determined by distance perception of individual places. Instead, spatial abilities of integrating separately learned places (Li and Giudice 2018) and understanding qualitative relations between them (Schwering et al. 2017) may play a more important role in effective spatial learning and successful wayfinding in a large-scale environment.

### Limitations

This study had several limitations that should be addressed in future studies. First, our measurement approach for both perceived and traversed distance relied on verbal reporting (participants provided an estimate in meters). Although we provided participants with a distance estimation training phase to familiarize them with estimating distance in this manner, not all dependent measurements such as blind-walking judgement and verbal reporting were equally affected by VR (Kelly et al. 2017; Philbeck et al. 2018). For example, Kelly et al. (2017) found that even though verbal reporting of perceived distance in VR was still underestimated, blind-walking judgements of perceived distance in VR and in the real world were comparable. In our study, we aimed to test distance perception in naturalistic conditions in both real-world and VR conditions, and this made some methods, such as blindfolding, less feasible to conduct in a public building. Second, in our examination of the effect of visual display (i.e., real-world walking versus VR-treadmill) on distance perception, not only the source of visual information was substituted but also proprioceptive input was potentially distorted. The ROVR treadmill provided certain proprioceptive experience which might still be substantially different from the real-world walking. In addition, treadmill walking does not provide the same vestibular input during translational movement in the real-world walking. This may also influence distance perception. Third, different size of the targets between the real world and VR might influence the comparison of perceived distance estimates between the real world and VR. We used a red board rather than a cylinder in the real world mainly due to that it was much easier to carry around a board than a cylinder. Although the width of the board was the same as the diameter of the cylinder in VR (1 m) and the board was held by an assistant at approximately the same height of the cylinder (3 m), the difference between targets might influence the comparison between perceived distance estimates in the real world and in VR. Nevertheless, the comparison between perceived distance estimation and actual distance was not affected.

## Conclusion

In this study, we set out to examine the effects of visual display, proprioception, and the continuity of optic flow on perceived, traversed, and environmental distance estimation in a large-scale building, by manipulating three popular virtual reality (VR) movement methods. Specifically, we compared emulated walking using a VR treadmill, moving with continuous optic flow using a touchpad, and moving with intermittent optic flow by teleportation. We then compared participants’ distance estimations with these three movement methods to their performance in the real world. The results showed that (1) visual display played a major role in the estimates of perceived and traversed distance, but proprioception and optic flow appeared not to affect these estimates, and (2) none of these three factors impacted the learning of environmental distance, and the accuracy of perceived and traversed distance estimates was not necessarily associated with the accuracy of environmental distance.

These findings have implications for spatial cognition research in large-scale virtual environments, as well as for other applications of VR such as architecture and urban planning. This study finds that, first, movement method does not influence people’s judgements of environmental distance ratio. Second, consistent with the literature, perceived and traversed distance judgements in VR are systematically underestimated. Future research in virtual and real environments should disentangle and examine the effects of environmental features (e.g., atriums) and social features (e.g., crowds) on distance perception and spatial cognition.

## Footnotes

1 https://github.com/stan-dev/stan/wiki/Prior-Choice-Recommendations
